# Improved frequency resolution for characterization of complex fractionated atrial electrograms

**DOI:** 10.1186/1475-925X-11-17

**Published:** 2012-04-03

**Authors:** Edward J Ciaccio, Angelo B Biviano, William Whang, Hasan Garan

**Affiliations:** 1 Department of Medicine, Division of Cardiology, Columbia University Medical Center, New York, NY, USA; 2 Columbia University, Harkness Pavilion 936, 180 Fort Washington Avenue, New York, NY, 10032, USA

**Keywords:** Atrial fibrillation, Ensemble averaging, Fourier analysis, Spectral estimation, Spectral resolution

## Abstract

**Background:**

The dominant frequency of the Fourier power spectrum is useful to analyze complex fractionated atrial electrograms (CFAE), but spectral resolution is limited and uniform from DC to the Nyquist frequency. Herein the spectral resolution of a recently described and relatively new spectral estimation technique is compared to the Fourier radix-2 implementation.

**Methods:**

In 10 paroxysmal and 10 persistent atrial fibrillation patients, 216 CFAE were acquired from the pulmonary vein ostia and left atrial free wall (977 Hz sampling rate, 8192 sample points, 8.4 s duration). With these parameter values, in the physiologic range of 3–10 Hz, two frequency components can theoretically be resolved at 0.24 Hz using Fourier analysis and at 0.10 Hz on average using the new technique. For testing, two closely-spaced periodic components were synthesized from two different CFAE recordings, and combined with two other CFAE recordings magnified 2×, that served as interference signals. The ability to resolve synthesized frequency components in the range 3–4 Hz, 4–5 Hz, …, 9–10 Hz was determined for 15 trials each (105 total).

**Results:**

With the added interference, frequency resolution averaged 0.29 ± 0.22 Hz for Fourier versus 0.16 ± 0.10 Hz for the new method (*p* < 0.001). The misalignment error of spectral peaks versus actual values was ±0.023 Hz for Fourier and ±0.009 Hz for the new method (*p* < 0.001). One or both synthesized peaks were lost in the noise floor 13/105 times using Fourier versus 4/105 times using the new method.

**Conclusions:**

Within the physiologically relevant frequency range for characterization of CFAE, the new method has approximately twice the spectral resolution of Fourier analysis, there is less error in estimating frequencies, and peaks appear more readily above the noise floor. Theoretically, when interference is not present, to resolve frequency components separated by 0.10 Hz using Fourier analysis would require an 18.2 s sequence duration, versus 8.4 s with the new method.

## Introduction

Accurate spectral resolution is important for analysis of atrial fibrillation (AF) in the frequency domain [1]. The information can be used, for example, to monitor periods of AF versus sinus rhythm in patients with paroxysmal arrhythmia. For understanding the mechanism by which the arrhythmia is maintained, it is also desirable to quantify any temporal variation in frequency that can occur during AF [2]. Since frequency shifts can be minute, sufficient resolving power is essential to detect these differences over short time intervals. Yet this becomes more difficult when signal-to-noise ratio is low, as is often the case. Another impediment to the generation of an accurate spectral profile is the presence of transients in the signal that are unrelated to physiologically relevant phenomena. One way to improve continuity in the frequency domain representation of AF from one instance of time to the next is to model and update the spectral profile based on new signal information as it is acquired in atrial fibrillation signals [2,3]. By using Gaussian functions to model the spectral peaks, after leveling the spectral baseline, spurious or transient changes to the signal frequency content can be excluded from the updates. When AF organization is reduced, the harmonic pattern is also expected to diminish [2], while independent spectral peaks caused for example by wavebreak can increase [4]. When resolving power is insufficient, a broad spectral peak in the Fourier power spectrum may thus be indicative of either a temporal variation in the frequency of a single component, or the merging of multi-component independent sources [5]. Therefore, the need for sufficient spectral resolution is essential for AF analysis. Improved resolution could also be useful to study the evolution and spontaneous termination of paroxysmal AF, as well as to estimate the organization of atrial activity [6].

In recent work we introduced a paradigm for spectral estimation and signal transformation of complex fractionated atrial electrograms (CFAE) based upon ensemble averaging [7-9]. This relatively new technique, like Fourier analysis, depends upon the autocorrelation function for generation of a power spectrum [9]. For spectral estimation, Fourier analysis models the sinusoidal properties of the autocorrelation function, while the new technique averages the autocorrelation function at lags w, 2w, 3w, …, where w is the period [9]. The spectral resolution of Fourier analysis is constant across bandwidth, while the spectral resolution of the ensemble average method depends inversely on w. The 1/w relationship results in greater spectral resolution at lower frequencies, which includes the electrophysiologic range of interest of ~ 3–10 Hz that is used for AF characterization. Herein, the new technique is compared to Fourier analysis for discerning closely-spaced frequency components in CFAE, which are of interest to characterize the arrhythmia and to identify dominant frequencies.

## Method

### Clinical data acquisition

Atrial electrograms were recorded in a series of 20 patients referred to the Columbia University Medical Center cardiac electrophysiology (EP) laboratory for catheter ablation of AF. These recordings were obtained prospectively as approved by the Internal Review Board at Columbia University Medical Center, but were analyzed retrospectively after the catheter ablation procedures were completed using standard clinical protocols. Ten patients had documented clinical paroxysmal AF, and all 10 had normal sinus rhythm as their baseline rhythm in the EP laboratory. AF was induced by burst atrial pacing from the coronary sinus or right atrial lateral wall, and persisted for at least 10 minutes for those signals included in the retrospective analysis of this study. Ten other patients had longstanding persistent AF, and had been in AF without interruption for 1–3 years prior to the catheter mapping and ablation procedure. The surface electro gram signals were acquired in analog form using the GE CardioLab system (GE Healthcare, Waukesha, WI) and filtered from 30-500 Hz with a single-pole band pass filter to remove baseline drift and high frequency noise. The filtered signals were digitally sampled by the system at 0.977 KHz and stored. Although the band pass high end was slightly above the Nyquist frequency, negligible signal energy is expected to reside in this frequency range [8].

Only signals identified as CFAEs by two cardiac electro physiologists were included in the retrospective analysis. Candidate CFAE recordings of at least 10 seconds in duration were obtained from two sites outside the ostia of each of the four pulmonary veins (PV). Similar recordings were obtained at two sites on the endocardial surface of the left atrial free wall, one in the mid-posterior wall, and another on the anterior ridge at the base of the left atrial appendage. From each of these recordings, 8.4-second sequences (8192 sample points) were analyzed. A total of 240 such sequences were acquired during electrophysiologic analysis – 120 from paroxysmal and 120 from longstanding AF patients. Subsequently, only 216 of the recordings were determined to be CFAE, and only these were used for subsequent analysis. As in previous studies, all CFAE signals were normalized to mean zero and unity variance prior to further processing [9].

### Frequency resolution

Using the radix-2 implementation for Fourier spectral estimation, the frequency resolution is:

(1) $$\begin{array}{c} {\text{R}}_{\text{F}}=\text{sample rate}/\#\;\text{discrete sample points}\\ =1/\text{time duration} \end{array}$$

This resolution is uniform throughout the frequency range. Based upon the Nyquist theorem, the range extends from DC to a maximum of ½ the sample rate. Atrial fibrillation signals are typically acquired at 977 Hz digitization, as was done in this study. For evaluation of the frequency content of CFAE, time durations of approximately 8 seconds are desirable for analysis [10]. Using these parameter values, the resolution would be:

(2) $${\text{R}}_{\text{F}}=(977\;\text{samples}/\text{s})/8192\;\text{samples}=0.12\;\text{Hz}$$

This frequency resolution is uniform throughout the range of DC to 977/2 = 489 Hz. The total number of data points in the resulting power spectrum will be 8192/2 = 4096. Thus the frequency resolution above the electrophysiologic range of interest, 10 Hz, to the high limit of 489 Hz, is the same as the frequency resolution within the physiologic range of 3–10 Hz.

A recently described technique that utilizes signal averaging was also used for spectral estimation [7-9]. Briefly, an ensemble average vector e_w_ with dimension w × 1 is obtained by averaging the n successive mean zero segments of a signal x having dimension N × 1, with each segment being of length w:

(3) $${\text{e}}_{\text{w}}=1/\text{n}\;U_{\text{w}}\text{x}\;$$

(4) $$U_{\text{w}}=\left[I_{\text{w}}I_{\text{w}}\ldots I_{\text{w}}\right]$$

Where an underline denotes a vector, bold font indicates a matrix, U_w_ is the summing matrix with dimension w × N, and I_w_ are w × w identity submatrices used to extract the signal segments from x. The number of signal segments in the summation is:

(5) $$\text{n}=\text{int}\left(\text{N}/\text{w}\right)$$

where int is the integer function. If N/w is not an integer, then in forming U_w_, 0’s are added to the N – (n · w) columns at the matrices’ right edge [9]. The segment length w can be converted to a frequency:

(6) f=sample rate/w

The power in the ensemble average is given by:

(7) $${\text{P}}_{\text{w}}=1/\text{w}\;{{\text{e}}_{\text{w}}}^{\text{T}}\cdot {\text{e}}_{\text{w}}$$

To construct the power spectrum, the root mean square (RMS) power can be used [7-9]:

(8) $${\text{P}}_{\text{wRMS}}=\sqrt{\left({\text{P}}_{\text{w}}\right)}$$

which has units of millivolts. The power spectrum can be displayed by plotting √n·P_wRMS_ versus frequency f. The √n term levels the spectral baseline, which would otherwise decrease by 1/√n, the noise falloff per number of summations n used for ensemble averaging. Computer code to calculate this spectrum has been presented and described elsewhere [9].

From Eq. 5, it is apparent that the frequency resolution of the new spectral estimation technique is proportional to 1/w. This resolution can be calculated as:

(9) $$\begin{array}{c} {\text{R}}_{\text{N}}=\left[\text{SR}/\text{w}\right]-\left[\text{SR}/\left(\text{w}+1\right)\right]\\ ={\text{f}}_{\text{w}}-{\text{f}}_{\text{w}+1} \end{array}$$

where R_N_ is the resolution of the spectral estimator, SR is the sample rate, and f_w_ and f_w+1_ are any two adjacent points on its frequency spectrum. The frequency resolution will be higher (i.e., the difference f_w_ − f_w+1_ will be smaller) at points along the spectrum where period w is longer (i.e. at low frequencies).

Akin to zero padding in Fourier analysis, it would be possible to interpolate between discrete sample points and use real values of w to boost the number of spectral points generated by the new method, but like padding for Fourier analysis, this would not add information to the system. The integer values of w range from 2 to N/2, where the upper bound of N/2 is given by the requirement that at least two signal segments are needed to compute an ensemble average. The value at w = 1 is the DC level. Thus analogous to the Fourier power spectrum, the power spectrum using the new technique also has 4096 points of resolution. Yet, unlike Fourier analysis, this resolution is not uniform. Based upon the 1/x relationship between frequency f and period w, the resolution will be poor at high frequencies but excellent at low frequencies. This property can be exploited for analysis of CFAE, since much of the frequency content of interest is in the range 3–10 Hz [7,8]. In this range the average frequency resolution of the new technique can be estimated from the resolution at representative frequency values. For example the resolution at 3.5 Hz is given by:

(10) $$\begin{array}{c} {\text{R}}_{\text{N},3.5\text{Hz}}=977/\text{w}-977/\left(\text{w}+1\right)\\ =977/279–977/280\\ =3.502\;\text{Hz}–3.489\;\text{Hz}=0.013\;\text{Hz} \end{array}$$

Similarly, the resolution at 4.5 Hz is:

(11) $$\begin{array}{c} {\text{R}}_{\text{N},4.5\text{Hz}}=977/\text{w}-977/\left(\text{w},+,1\right)\\ =977/217–977/218\\ =4.502\;\text{Hz}–4.481\;\text{Hz}=0.021\;\text{Hz} \end{array}$$

The average resolution as determined from the resolution at 3.5, 4.5, 5.5,…, 9.5 Hz is 0.05 Hz. However to resolve two peaks, there must be at least one point of power spectral resolution separating them. Thus the minimum frequency at which two spectral peaks can be resolved would be 2 × 0.12 Hz = 0.24 Hz for Fourier, and on the average 2 × 0.05 Hz = 0.10 Hz for the new method in the range 3–10 Hz (see second column, Table 1). As a note of comparison, to theoretically resolve frequency components separated by 0.10 Hz using Fourier analysis would therefore require an 18.2 s sequence duration, versus 8.4 s with the new method.

**Table 1:** Resolution to discern closely-spaced frequency components

**Freq. (Hz)**	**Res-theor (Hz)**	**Res-NT (Hz)**	**Res-FT (Hz)**	**Significance**
9.37 ± 0.47	0.19	0.21 ± 0.05	0.25 ± 0.08	P = 0.307
8.49 ± 0.24	0.15	0.23 ± 0.09	0.28 ± 0.08	P = 0.105
7.51 ± 0.32	0.11	0.18 ± 0.07	0.29 ± 0.18	P = 0.081
6.47 ± 0.27	0.09	0.14 ± 0.04	0.32 ± 0.20	P = 0.003
5.45 ± 0.32	0.06	0.13 ± 0.09	0.27 ± 0.18	P < 0.001
4.37 ± 0.27	0.04	0.08 ± 0.03	0.22 ± 0.07	P < 0.001
3.51 ± 0.29	0.03	0.07 ± 0.04	0.19 ± 0.06	P < 0.001
6.41 ± 2.01 (MN)	0.10	0.16 ± 0.10 (MN)	0.29 ± 0.22 (MN)	P < 0.001

Freq.: the mean frequency used for the test, Res-theor – the theoretical resolving power of the new method, Res – NT: the resolving power of the new method, Res – FT: the resolving power of Fourier analysis, Significance – the significance of the difference between the resolving power of the techniques based on the unpaired *t*-test. MN – mean value.

### Synthesis of simulated CFAE and power spectra

CFAE with closely-spaced frequency components were artificially synthesized to test the resolution of the methods as follows. First a sequence of random length ω was extracted from one of the 216 CFAE recordings, which was also selected at random. The extracted sequence of length ω was repeated to N = 8192 sample points to form one signal component. Then another random sequence, with length ω + 2, was extracted from another randomly selected CFAE recording. The extracted sequence of length ω + 2 was also repeated to N = 8192 sample points to form a second signal component. Two other of the 216 CFAE recordings selected at random were each amplified with a gain of ×2 to be used as interference as in a previous study [8]. Thus the following four randomly obtained sequences of length 8192 sample points were summed and used for subsequent analysis:

1. signal component 1 with repeating period w

2. signal component 2 with repeating period w + 2

3. interference 1 with 2× gain

4. interference 2 with 2× gain

**Figure 1:**
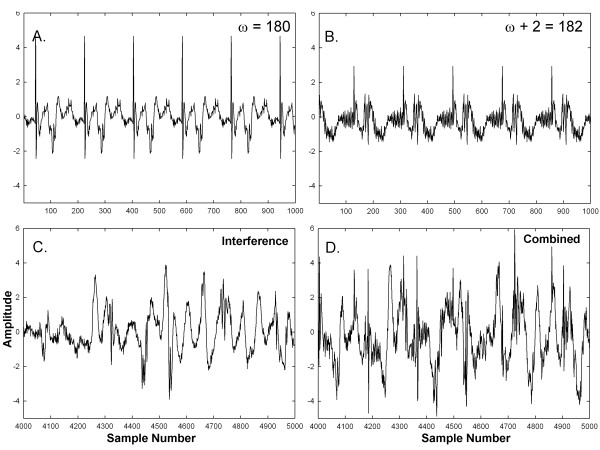
**Example of synthesized signals at the low range in frequency.A-B**. Synthesized frequency components. **C**. Interferences. **D**. Combination of synthesized frequency components and interferences.

**Figure 2:**
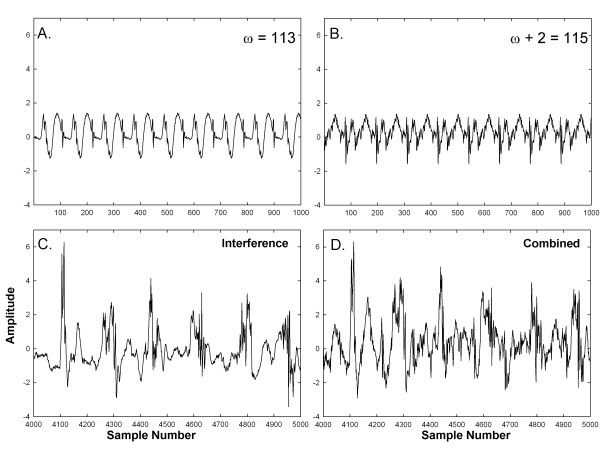
**Example of synthesized signals at the high range in frequency.A-B**. Synthesized frequency components. **C**. Interferences. **D**. Combination of synthesized frequency components and interferences.

An example of the synthesis process is shown in Figure 1 for frequencies toward the low end of the spectral range. Repeating segments with ω = 180 sample points and ω + 2 = 182 sample points are shown in panels A and B, respectively (f = 5.43 Hz and 5.37 Hz). The sum of two additive interferences with a gain of 2× is shown in panel C. The result from combining the components of panels A, B, and C is shown in panel D. The original periodicities are mostly unrecognizable in panel D. Similarly, an example for components with frequencies toward the high end of the electrophysiologic range of interest is shown in Figure 2. Repeating segments with ω = 113 sample points and ω + 2 = 115 sample points are shown in panels A and B, respectively (f = 8.65 Hz and 8.50 Hz). Additive interferences are shown in panel C. The result from combining the components of panels A, B, and C is shown in panel D. The original periodicities are again mostly unrecognizable.

This paradigm was repeated so that a total of 15 simulated CFAE with closely-spaced frequency components in the range 3–4 Hz, 15 in the range 4–5 Hz, …, and 15 in the range 9–10 Hz were constructed. Thus the total number of simulation signals to be analyzed, of the type shown in Figure 1D and 2D, was 7 × 15 = 105.

Prior to Fourier analysis, each simulated signal was zero-padded to a length N = 65536, for a final interval of 0.015 Hz between spectral points. By zero padding, the frequency resolution of the FFT was not increased, only the point-to-point interval in the resulting spectrum, because the length of the observed signal was unchanged. Zero padding ensured that closely-spaced spectral components would be evident within the limit given by the frequency resolution constraint. The Fourier power spectrum was generated using a radix-2 implementation [11]. Initially the simulated CFAE were preprocessed with a Han window prior to Fourier analysis. However, while Han windowing can improve periodicity by forcing the signal ends to zero, it also adds distortion in the form of amplitude modulation. The amplitude modulation can impart spectral sidebands which reduce frequency resolution. It was also observed visually that discernment of closely-spaced frequency components was improved without this filter. Therefore a rectangular window (i.e. no filter) was used for Fourier analysis. Likewise, no window preprocessing was done prior to spectral estimation with the new method.

**Figure 3:**
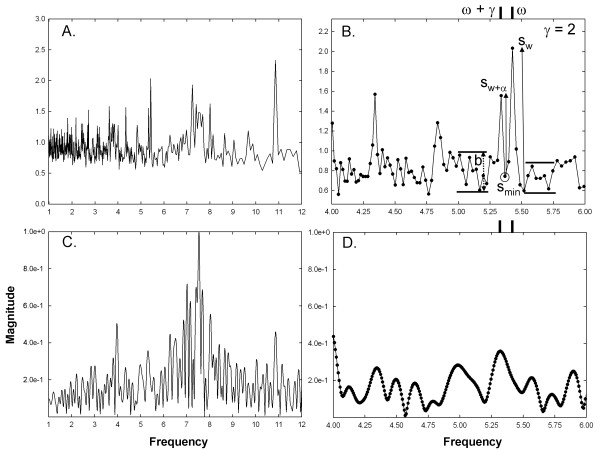
**Examples of frequency spectra used for analysis for CFAE with two closely-spaced frequency components at the low end of range. A**. The power spectrum using the new technique in the range 1–12 Hz. **B. A** close-up of the power spectrum with a range of 2 Hz. The parameters used to determine whether the synthetic frequency components are resolved are shown. **C**. The Fourier power spectrum in the range 1–12 Hz. **D**. A close-up of the Fourier power spectrum with a range of 2 Hz. The synthetic frequency components are not resolved in the Fourier spectrum.

Consider the spectral profiles of both Fourier and new technique, each of which consists of 4096 data points for parameter values of 977 Hz sample rate and a signal length of 8192 sample points. As shown in Figures 1 and 2, the initially synthesized signals had frequency components of ω and ω + γ, where γ = 2, the minimum resolving distance. To determine the ability of each method to resolve closely-spaced peaks, γ was increased by 1 in each of the simulated signals until two distinct peaks appeared in the spectrum. Closely-spaced synthesized frequency components were considered to be resolved for the minimum value of γ for which any resulting spectral peaks with periods w and w + α met the following four criteria. The first requirement for discernability was that:

(12) $${\text{s}}_{2}>{\text{s}}_{1}/4$$

where s is the amplitude of the tallest spectral peak in proximity to the actual location of a synthesized frequency component, referenced to that foot which gives the largest peak amplitude, s_1_ is the amplitude of the higher peak, and s_2_ is the amplitude of the lower peak. An example of peak amplitude measurement is shown in Figure 3B (solid vertical arrows). The second requirement for discernability is that the minimum value between the two peaks:

(13) $${\text{s}}_{\text{min}}\text{=min}\left[{\text{s}}_{\text{w+1}}:{\text{s}}_{\text{w+α−1}}\right]$$

is within or below the maximum range b of the neighboring background level (Figure 3B). The neighboring background level is defined as the range in magnitude −0.25 Hz in frequency away from the left foot of peak w + α, and +0.25 Hz in frequency away from the right foot of peak w (horizontal lines, Figure 3B). The third criterion is that:

(14) $${\text{s}}_{\text{w}},{\text{s}}_{\text{w}\text{+α}}>\text{b}$$

that is, the magnitudes s_w_ and s_w+α_ are greater than the maximum range b of the neighboring background level (Figure 3B). The final criterion is that the peaks at w and w + α are each within ±0.15 Hz of the two synthesized frequency components having actual periods of ω and ω + γ (noted with bars at the top of the graph in Figure 3B).

The spectral resolution of Fourier analysis versus the new technique in the presence of interference were determined as follows. The criteria as given by Equations 11, 12 and 13 were tested for two synthesized spectral components with periods ω and ω + γ, γ = 2 through 20 spectral points. Again, the lower limit of 2 was necessitated by the fact that two frequency components occurring at adjacent spectral points (γ = 1) cannot be resolved. The minimum value of γ for which these criteria were met was defined as the spectral resolution to discern the closely-spaced simulated spectral peaks. If these criteria were not met to γ = 20, the trial was tabulated as being one in which the resolution was indeterminable due to the loss of one or more spectral peaks in the noise floor. For both methods, the estimate error was defined as the average of the absolute difference in f_w_ - f_ω_ and f_w+α_ – f_ω+γ_, where f_w_ and f_ω_ are the measured and actual frequencies of one of the peaks, and f_w+α_ and f_ω+γ_ are the measured and actual frequencies of the other peak, respectively. For Fourier analysis, a correction was made by subtracting 0.06 Hz from each error after calculating the absolute difference of measured to actual frequency, with the minimum error after subtraction being 0.00 Hz. This allowed for the fact that for simplicity, the synthetic frequencies that were used were always 1/w, with w being an integer, so that on average for Fourier analysis, having constant increments of 0.12 Hz between spectral points, the actual frequency components could be different by as much as ±0.06 Hz. For the new technique, the spectral points occurred at 1/w for all w in range, so no correction was done.

The overall paradigm was repeated for 15 trials in each frequency range 3–4 Hz, 4–5 Hz, …, 9–10 Hz for both Fourier analysis and the new technique. The results were averaged and tabulated as mean ± standard deviation. The unpaired *t*-test was used to determine the significance of the difference between methods (SigmaPlot 2004 for Windows Ver. 9.01, Systat Software, Chicago, and MedCalc ver. 9.5, 2008, MedCalc Software bvba, Mariakerke, Belgium).

## Results

### Spectral properties

**Figure 4:**
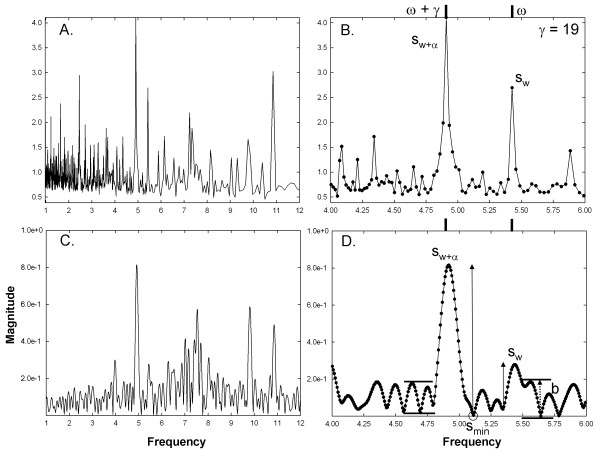
**Same data as in Figure**3**except that the synthetic frequency components are spaced further apart (γ is larger).** The panels correspond to those in Figure 3. **B**. The synthetic frequency components remain resolved using the new technique. **D**. The synthetic frequency components resolve using Fourier analysis.

A power spectrum using the new spectral estimation technique is shown in Figure 3A. Note that the highest frequency resolution occurs at lower frequencies due to the 1/w relationship of resolution to frequency for this method. By comparison, the Fourier power spectrum is uniform in resolution across the range (Figure 3C). Figures 3B and D show close-ups of the respective spectra in the range of the synthesized components. The actual synthesized components have frequencies of 5.34 Hz (ω + γ = 183 sample points at 977 Hz sampling rate) and 5.43 Hz (ω = 180 sample points), noted by vertical bars at the tops of panels 3B and 3D. The two components are correctly resolved by the new technique (panel 3B), that is, w = ω and w + ∝ = ω + γ. However, Fourier analysis does not resolve at this component spacing (panel 3D). In Figure 4, using the same CFAE and with the high frequency remaining at 5.43 Hz (ω = 180 sample points), the result is shown for γ = 19 when the low frequency is 4.91 Hz (ω + γ = 199 sample points). The spectrum and close-up using the new method are shown in Figure 4A-B, and the frequency components are readily resolved as in Figure 3A-B. The Fourier spectrum is shown in Figure 4 C-D and now distinct peaks appear (Figure 4D), meeting the criteria set forth in the Methods. This was the minimum distance γ at which two corresponding Fourier spectral peaks met the criteria, and therefore the resolution for the Fourier spectrum. The measurements for s_w+α_, s_w_, s_min_, and b are shown.

**Figure 5:**
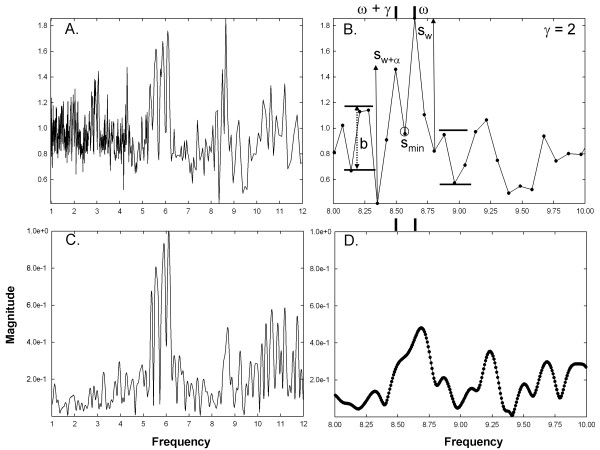
**Examples of frequency spectra used for analysis for CFAE with two closely-spaced frequency components at the high end of range.** Panels correspond to those in Figure 3. The synthetic frequency components reside at approximately 8.5 Hz, and are resolved by the new technique (panel B). Measurement parameters are shown. The synthetic frequency components are not resolved by Fourier analysis (panel D).

**Figure 6:**
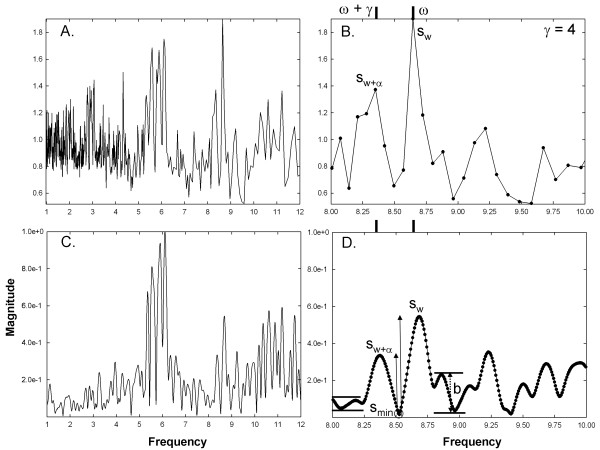
**Same data as in Figure**5**except that the synthetic frequency components are spaced further apart.** The panels correspond to those in Figure 3. The synthetic frequency components remain resolved using the new technique in panel B. The synthetic frequency components resolve using Fourier analysis in panel D.

The power spectrum from 1–12 Hz using the new technique with a different set of signals, and synthesized components near the higher end of the frequency range is shown in Figure 5A. The components reside at f_ω+γ_ = 8.50 Hz and f_ω_ = 8.65 Hz. Several subharmonics present in the range 1–4 Hz are evident in Figure 5A. The Fourier power spectrum from the same synthesized signal is shown in the range 1–12 Hz in Figure 5C. There are substantial superharmonics in the high frequency range from 9.5–12 Hz. Close-ups are shown in the right-hand panels for both methods. Even though the spectral resolution for the new technique is diminished in this frequency range, it resolves the peaks when γ = 2 (Figure 5B). The parameters used for measurement are shown in this panel. The Fourier power spectrum does not resolve at this level (panel 5D). There is only a single blunted peak from about 8.5 Hz to 8.75 Hz. The same data is shown for γ = 4 in Figure 6. The frequency components remain involved for the new method (Figure 6B) and first resolve for Fourier analysis (Figure 6D). The actual frequency components have values of f_ω+γ_ = 8.35 Hz and f_ω_ = 8.65 Hz. The parameters indicating that the Fourier spectral peaks resolve are noted (Figure 6D).

### Summary statistics

For a total of 105 trials, excluding those in which the synthesized components could not be resolved, the mean frequency resolution with the presence of additive interference was 0.29 ± 0.22 Hz for Fourier and 0.16 ± 0.10 Hz for the new technique (*p* < 0.001). Thus the resolving power of each method, in the presence of interference, was similar to their respective theoretical resolutions of 0.10 Hz and 0.24 Hz. A complete summary of the resolving power of both techniques is provided in Table 1. The mean frequency for the random trials in each frequency bin that resolved are shown in the rows of column 1 from top to bottom (9–10 Hz, 8–9 Hz, …). The expected resolution at 9.5 Hz, 8.5 Hz, …, using the new method, are noted in the second column. The actual resolving power for the new technique and for the Fourier transform are shown in the next two columns, respectively. At all frequencies, the new technique resolving power is improved over the Fourier method. The significance of the difference in mean resolving power between the methods is noted in the right-hand column. At lower frequencies up to 7 Hz there is a highly significant difference (*p* ≤ 0.003). At higher frequencies the difference trends toward significance.

The average of the absolute misalignment of spectral peaks with actual values of synthesized components (the estimate error) was 0.023 ± 0.039 Hz for Fourier and 0.009 ± 0.030 Hz for the new technique (*p* < 0.001). A complete summary of the estimate error for both methods is provided in Table 2. The mean frequency for the trials in each frequency bin that resolved is again shown in the rows of column 1 from top to bottom, as in Table 1. The estimate error values for each frequency bin are shown for the new technique and for Fourier analysis in the middle columns. In each case, the error, or misalignment of spectral peak with respect to actual component frequencies, is substantially less using the new technique. The significance of the difference in error between the new technique and Fourier analysis is noted in the right-hand column. There is a high degree of significance at all frequencies except the 9–10 Hz bin.

**Table 2:** Estimate error in the frequency of peak values

**Freq. (Hz)**	**Error NT (Hz)**	**Error FT (Hz)**	**Significance**
9.37 ± 0.47	0.032 ± 0.074	0.039 ± 0.070	P = 0.708
8.49 ± 0.24	0.004 ± 0.003	0.016 ± 0.015	P < 0.001
7.51 ± 0.32	0.004 ± 0.002	0.027 ± 0.043	P = 0.006
6.47 ± 0.27	0.005 ± 0.007	0.017 ± 0.023	P = 0.011
5.45 ± 0.32	0.003 ± 0.002	0.018 ± 0.023	P = 0.001
4.37 ± 0.27	0.003 ± 0.002	0.019 ± 0.019	P < 0.001
3.51 ± 0.29	0.013 ± 0.035	0.043 ± 0.053	P = 0.016
6.41 ± 2.01 (MN)	0.009 ± 0.030 (MN)	0.023 ± 0.039 (MN)	P < 0.001

Freq.: the mean frequency used for the test, Error – NT: the difference between the actual and estimated frequency using the new method, Error – FT: the absolute difference between actual and estimated frequency using Fourier analysis, Significance – the significance of the difference between the techniques based on the unpaired *t*-test. MN – mean value.

Overall, of 15 trials for each frequency bin (105 trials in all), one or both frequency components did not appear above the noise floor in 13/105 trials using the Fourier method (12.4%) versus 4/105 trials using the new technique (3.8%).

## Discussion

### Summary

In this study a comparison was made between the ability to resolve two closely-spaced frequency components in the physiologic range of interest using Fourier power spectral analysis, versus a new technique that utilizes signal averaging. The synthesized closely-spaced frequency components and two additive interferences were selected at random from a set of 216 CFAE. The values for digital sampling rate (977 Hz) and sequence length (N = 8192, 8.4 s sequences) are typical of those used for frequency analysis of CFAE obtained during clinical EP study. Tests were made in the range 3–10 Hz, the electrophysiologic range for evaluation of atrial electrical activity. From 105 tests, the mean resolving power of Fourier versus the new technique (0.29 Hz versus 0.16 Hz; *p* < 0.001), were higher than the theoretical values but in accord with the presence of large interferences that could act to mask the frequency components. In 13/105 trials, interference masked frequency components in the Fourier power spectrum. By comparison, this occurred in only 4/105 trials using the new technique. The error in estimating the synthesized components was ±0.023 Hz using Fourier versus ±0.009 Hz using the new technique (*p* < 0.001).

### Signal segment not used for spectral estimation

**Figure 7:**
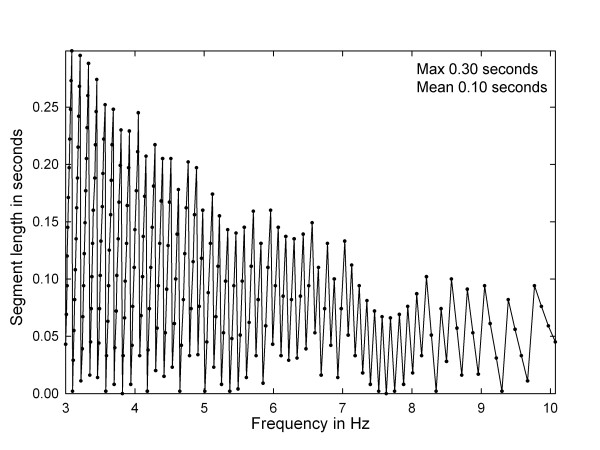
**Plot of the segment length at the end of the each CFAE signal that is unused for each power spectrum calculation using the new technique, versus frequency in the physiologic range**.

Based upon Eq. 4, whenever n is not an integer, a portion of the 8.4 second CFAE signal at its end is not used for spectral estimation. This can be described as:

(15) $$\text{L}=\left[\text{N}/\text{w}-\text{int}\left(\text{N}/\text{w}\right)\right]/977$$

where L is the unused signal segment length in seconds. In Figure 7 is shown a plot of segment length versus frequency in the physiologic range of interest (3–10 Hz). The maximum unused segment length, 0.3 seconds, occurs at 3.1 Hz, near the lowest frequency in the range. The mean unused segment length is approximately 0.1 seconds. Thus on the average, 8.3 seconds of the 8.4 second signal is used for power spectral estimation, meaning that only about 1% of the signal is not included in the measurement.

### Clinical correlates

The dynamics of internal fibrillatory activity are of great interest to establish AF mechanisms as well as for catheter ablation, and can be measured using markers of organization [12]. It would be desirable to correlate these invasive measurements with noninvasive electrocardiogram measurements, to determine the extent to which AF can be characterized noninvasively with for example, a Holter monitor [12]. It is also desirable to distinguish slightly different levels of organization during AF by improving temporal resolution and sensitivity [13]. Such improvements could lead to new ways of analyzing and understanding AF, and improved AF treatment methods [13]. The parameters used in our study for analysis of CFAE are typical for clinical investigations. The capability of the Fourier transform to resolve two independent frequency components in close proximity, 0.24 Hz, is unacceptable for describing the temporal evolution of paroxysmal AF or to differentiate this type from persistent AF. Using Fourier analysis, the dominant frequency, defined as the tallest spectral peak in the physiologic range of interest, often varies with a standard deviation of 0.2–0.3 Hz when CFAE are recorded from each of the four pulmonary vein ostia [14]. Thus the resolving power using the Fourier spectrum is likely to be insufficient for detailed, accurate measurement. To determine whether differences in the frequencies of spectral peaks are real or artifact requires an increase in resolving power, as is possible with our new technique. Furthermore, the ability to resolve multiple characteristic frequencies in AF signals, which may be caused by multiple wavelets propagation as are commonly found in AF data, is more difficult when the electrical activity is highly unorganized [15]. Applying the new technique to these signals can potentially increase the ability to spectrally resolve the multiple activation wavelets that can be present in the arrhythmogenic substrate.

## Conclusions

In this study we demonstrated that the resolving power to discern two closely-spaced synthesized frequency components was significantly greater using a new spectral estimation technique as compared with Fourier analysis. Furthermore, the new technique estimates the actual frequencies of the components to a significantly greater degree of accuracy as compared with Fourier. The simulations suggest that two closely-spaced frequency components, as might be encountered by drivers of electrical activation in close spatial proximity to one another within the arrhythmogenic substrate that is causing atrial fibrillation, can be discerned by the new technique, even in combination with substantial interference levels. Such interferences may represent, for example, independent drivers at the periphery of the substrate that are inconsequential to the maintenance of AF. Thus this method may be helpful to detect and identify independent drivers in the atrial substrate that can be masked by interference from bystander regions. Such information is potentially important to develop a mechanistic understanding of the pattern of electrical activation during atrial fibrillation. Although only CFAE simulations were done in this study, these observations are probably applicable to other types of signals including ventricular electrograms [16] and electrocardiogram data [17]. Furthermore, better spectral resolution can be important for improved recognition of repeating patterns in CFAE when analyzed in the frequency domain [18].

### Limitations

For simplicity, the number of trials for discerning closely-spaced frequency components was limited to 15 for each of seven frequency bins in the electrophysiologic range of interest. The types of interference signals and the types of periodic components that were used were limited to those present in a pool of 216 CFAE recordings obtained from the pulmonary vein ostia and left atrial free wall in both paroxysmal and longstanding AF patients. The use of synthesized components was necessitated by the fact that frequency values needed to be known with certainty. Since synthesized signals were tested, they will not necessarily represent the performance of Fourier spectral analysis versus the new technique on real data. Still, since the synthesized frequency components and interferences were extracted from real CFAE, we believe that they are representative of the types of signals that would be encountered in real measured data.

## Competing interests

The authors declare that they have no competing interests.

## Author contributions

EJC developed the mathematical methods, conducted the data analysis, and wrote the manuscript. ABB, WW, and HG made helpful suggestions, provided the clinical data, and determined which recordings were complex fractionated atrial electrograms. All authors have read and approved the final manuscript.
